# Glucose-Dependent Insulinotropic Polypeptide Inhibits AGE-Induced NADPH Oxidase-Derived Oxidative Stress Generation and Foam Cell Formation in Macrophages Partly via AMPK Activation †

**DOI:** 10.3390/ijms25179724

**Published:** 2024-09-08

**Authors:** Michishige Terasaki, Hironori Yashima, Yusaku Mori, Tomomi Saito, Naoto Inoue, Takanori Matsui, Naoya Osaka, Tomoki Fujikawa, Makoto Ohara, Sho-ichi Yamagishi

**Affiliations:** 1 Division of Diabetes, Metabolism, and Endocrinology, Department of Medicine, Graduate School of Medicine, Showa University, 1-5-8 Shinagawa, Tokyo 142-8666, Japan; yashima@med.showa-u.ac.jp (H.Y.); saito_to@cnt.showa-u.ac.jp (T.S.); gm23-n007@med.showa-u.ac.jp (N.I.); n.oosaka@med.showa-u.ac.jp (N.O.); ftlilac@med.showa-u.ac.jp (T.F.); s6018@med.showa-u.ac.jp (M.O.); shoichi@med.showa-u.ac.jp (S.-i.Y.); 2 Anti-Glycation Research Section, Division of Diabetes, Metabolism, and Endocrinology, Department of Medicine, Graduate School of Medicine, Showa University, 1-5-8 Shinagawa, Tokyo 142-8666, Japan; u-mori@med.showa-u.ac.jp; 3 Department of Bioscience and Biotechnology, Fukui Prefectural University, Eiheiji, Fukui 910-1195, Japan; matsuita@fpu.ac.jp

**Keywords:** GIP, AGEs, Cdk5, CD36, AMPK, NADPH oxidase

## Abstract

Glucose-dependent insulinotropic polypeptide (GIP) of the incretin group has been shown to exert pleiotropic actions. There is growing evidence that advanced glycation end products (AGEs), senescent macromolecules formed at an accelerated rate under chronic hyperglycemic conditions, play a role in the pathogenesis of atherosclerotic cardiovascular disease in diabetes. However, whether and how GIP could inhibit the AGE-induced foam cell formation of macrophages, an initial step of atherosclerosis remains to be elucidated. In this study, we address these issues. We found that AGEs increased oxidized low-density-lipoprotein uptake into reactive oxygen species (ROS) generation and *Cdk5* and *CD36* gene expressions in human U937 macrophages, all of which were significantly blocked by [D-Ala^2^]GIP(1–42) or an inhibitor of NADPH oxidase activity. An inhibitor of AMP-activated protein kinase (AMPK) attenuated all of the beneficial effects of [D-Ala^2^]GIP(1–42) on AGE-exposed U937 macrophages, whereas an activator of AMPK mimicked the effects of [D-Ala^2^]GIP(1–42) on foam cell formation, ROS generation, and *Cdk5* and *CD36* gene expressions in macrophages. The present study suggests that [D-Ala^2^]GIP(1–42) could inhibit the AGE-RAGE-induced, NADPH oxidase-derived oxidative stress generation in U937 macrophages via AMPK activation and subsequently suppress macrophage foam cell formation by reducing the Cdk5-CD36 pathway.

## 1. Introduction

Glucose-dependent insulinotropic polypeptide (GIP), a 42-amino-acid hormone, is one of the incretins released from K-cells in the small intestine with response to the ingestion of nutrients, such as sugar and fat and has been revealed to promote insulin secretion in a glucose-dependent manner [1,2,3,4]. Because of its insulin-secreting property, GIP could be a potential therapeutic strategy for the treatment of type 2 diabetes patients [5,6]. The biological effects of GIP on β-cells in the pancreas are mainly regulated by the high-affinity receptor for GIP (GIPR) [7,8] and a novel dual GIP and glucagon-like peptide-1 (GLP-1) receptor agonist, tirzepatide, has been developed for the treatment of patients with type 2 diabetes [9,10,11]. Since the GIP receptor is expressed in numerous non-pancreatic tissues, including vasculature, blood cells, adipose tissues, and the brain [12,13], GIP may have pleiotropic effects on the cardiovascular system [14,15,16,17]. Indeed, we have previously shown that the continuous infusion of active GIP(1–42) attenuates the progression of macrophage-derived atherosclerosis in the aortae of both diabetic and non-diabetic apolipoprotein E-null (*Apoe*^−/−^) mice, whose effects were independent of food intake, body weight, blood pressure, and plasma glucose or lipid levels [14]. Furthermore, in vivo-treatment with GIP inhibits the foam cell formation of macrophages extracted from diabetic and non-diabetic apolipoprotein E-null (*Apoe*^−/−^) mice [14]. In addition, we recently found that although foam cell formation was inhibited when macrophages were extracted from *Gipr*(^+/+^) mice subcutaneously infused with a dipeptidyl peptidase-4-resistant GIP analog, [D-Ala^2^]GIP(1–42), it was not observed in macrophages from similarly treated GIP receptor-deficient (*Gipr*^−/−^) mice [18]. These findings suggest that GIP may play a protective role against atherosclerotic cardiovascular disease by suppressing macrophage foam cell formation via the GIP receptor.

According to the report of the International Diabetes Federation Diabetes Atlas in 2021, the number of patients with diabetes aged 20–79 is increasing worldwide and is estimated to be 537 million. Diabetes mellitus accelerates the progression of atherosclerosis and the macrovascular complications of diabetes, such as coronary heart disease, peripheral artery disease, and stroke, which are a leading cause of death in patients with diabetes; about 50% of patients with diabetes mellitus die from atherosclerotic cardiovascular disease [19,20]. Various biochemical pathways evoked by diabetic conditions could contribute to the progression of atherosclerosis [21,22]. Among them, advanced glycation end products (AGEs), whose formation and storage are enhanced under chronic hyperglycemic and oxidative stress conditions [23,24,25], have been known to play a principal role in the pathogenesis of atherosclerotic cardiovascular disease [26,27,28]. AGEs have been reported to stimulate oxidative stress generation, inflammatory responses, and thrombotic reactions in various types of cells and tissues, including macrophages, via engagement with their cell surface receptor for AGEs (RAGEs) [23,29]. Indeed, AGEs are localized in macrophage-derived foam cells within atherosclerotic plaque lesions and could induce the foam cell formation of macrophages, thereby showing their involvement in atherosclerotic plaque instability and the resultant increase in the risk of atherosclerotic cardiovascular disease in diabetes patients [30,31,32,33,34]. Moreover, circulating and tissue-accumulated levels of AGEs are a biomarker to predict future cardiovascular events and death in patients with diabetes, thus suggesting that the blockade of the AGE-RAGE axis in macrophages is a novel therapeutic target for preventing atherosclerotic cardiovascular disease in diabetes [35,36,37,38]. However, whether and how GIP could inhibit the AGE-induced foam cell formation of macrophages remains to be elucidated. Therefore, in this study, we investigated the effect of [D-Ala^2^]GIP(1–42) on the foam cell formation of macrophages exposed to AGEs by evaluating oxidized low-density-lipoprotein (ox-LDL) uptake into AGE-exposed human U937 macrophages and further examining the underlying molecular mechanism for the anti-atherosclerotic effects of [D-Ala^2^]GIP(1–42) on macrophages.

## 2. Results

### 2.1. Gene Expression of Gipr and Rage in Human Monocyte-Derived U937 Cells

**Figure 1:**
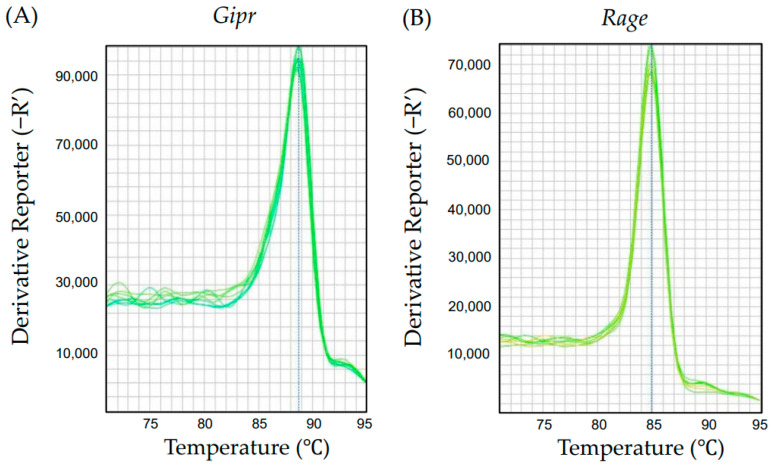
Melt curve of gene expression of *Gipr* (**A**) and *Rage* (**B**) in human U937 cells.

We investigated whether the *Gipr* and *Rage* genes were expressed in human U937 macrophages. As shown in Figure 1, the *Gipr* (A) and *Rage* (B) genes were actually expressed in U937 cells.

### 2.2. [D-Ala^2^]GIP(1–42) Inhibited the AGE-Induced Foam Cell Formation of and Intracellular Reactive Oxygen Species (ROS) Generation in Human U937 Cells

**Figure 2:**
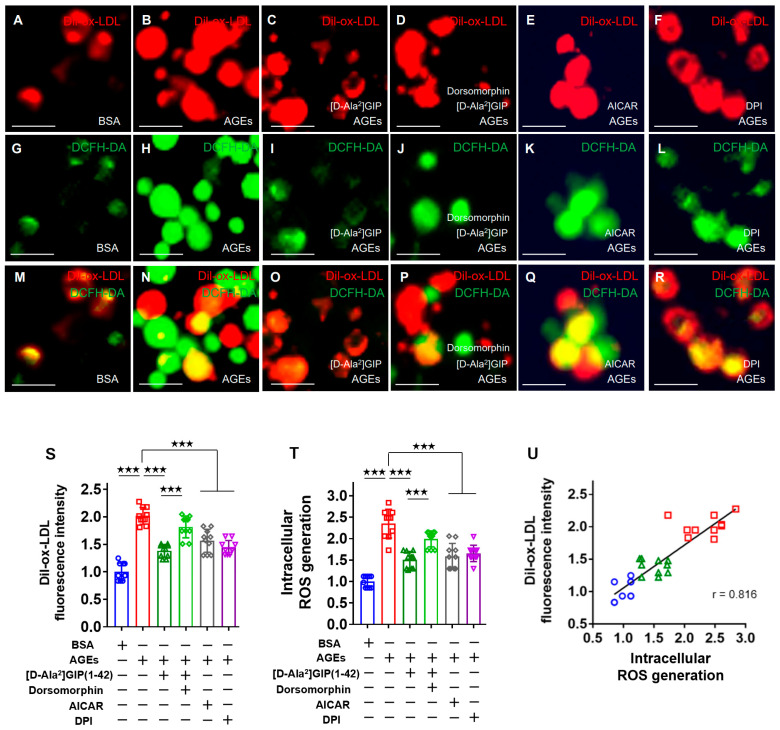
Effects of [D-Ala^2^]GIP(1–42), dorsomorphin, AICAR, and DPI on Dil-ox-LDL uptake into, and ROS generation in, AGE-exposed U937 macrophages. U937 cells were treated with 100 μg/mL AGE-BSA or 100 μg/mL non-glycated control BSA in the presence or absence of 1 nmol/L [D-Ala^2^]GIP(1–42), 50 nmol/L dorsomorphin, 500 μmol/L AICAR, or 50 nmol/L DPI in Roswell Park Memorial Institute (RPMI) 1640 medium supplemented with 10% fetal bovine serum (FBS) at 37 °C for 24 h. Then, the adherent cells were incubated with 10 μg/mL Dil-ox-LDL for 18 h and further treated with a 10 μmol/L DCFH-DA probe for another 15 min. The fluorescence intensity of Dil-ox-LDL and DCF were measured. (**A**–**R**) Representative images of immunofluorescence staining. Dil-ox-LDL-positive cells were stained in red, and ROS-generating cells are represented in green. Scale bars show 20 μm. (**S**–**U**) Quantitative data of fluorescence intensity for Dil-ox-LDL and DCF were normalized for each control value with BSA-treated cells, respectively. The correlation of fluorescence intensity between Dil-ox-LDL and DCF was used by Pearson’s correlation test. Number = 10 for each group. Values are shown as mean ± standard deviation. ★★★ *p* < 0.005. (**U**) Blue circles for BSA-treated cells, red squares for AGE-treated cells, and green triangles for AGE-treated cells with [D-Ala^2^]GIP(1–42).

We first investigated the effect of [D-Ala^2^]GIP(1–42) on the AGE-induced foam cell formation of macrophages. Macrophage foam cell formation was evaluated by 1,1-dioctadecyl-3,3,3,3-tetamethylindocarbocyanine perchlorate (Dil)-labeled oxidized-LDL (Dil-ox-LDL) uptake into U937 cells. As shown in Figure 2, immunofluorescence staining showed that [D-Ala^2^]GIP(1–42) significantly inhibited the increase in Dil-ox-LDL uptake into AGE-exposed U937 cells, whose effect was attenuated by dorsomorphin, which is an inhibitor of AMP-activated protein kinase (AMPK). Furthermore, an activator of AMPK, 5-aminoimidazole-4-carboxamide1-B-D-ribofuranoside (AICAR), mimicked the inhibitory effect of [D-Ala^2^]GIP(1–42) on macrophage foam cell formation, while an inhibitor of NADPH oxidase, diphenylene iodonium (DPI), significantly inhibited the stimulatory effect of AGEs on Dil-ox-LDL uptake into U937 cells.

When intracellular oxygen species (ROS) generation was measured by fluorescence intensity of 2′,7′-dichlorodihydrofluorescein (DCF) using a cell-permeable probe, 2′,7′-dichlorodihydrofluorescein diacetate (DCFH-DA), as in the case of the foam cell formation of U937 cells, AGEs increased ROS generation in U937 cells, which was significantly inhibited by [D-Ala^2^]GIP(1–42) or DPI. The anti-oxidative effect of [D-Ala^2^]GIP(1–42) on AGE-exposed U937 cells was partially attenuated by co-treatment with dorsomorphin, while AICAR mimicked the effect of [D-Ala^2^]GIP(1–42) on ROS generation in U937 cells exposed to AGEs.

As shown in Figure 2, some Dil-ox-LDL-positive cells had DCF intensity. There was a significant correlation of fluorescence intensity between Dil-ox-LDL and DCF, thus suggesting that intracellular ROS generation occurred in Dil-ox-LDL-uptake macrophages (Figure 2U).

### 2.3. [D-Ala^2^]GIP(1–42) Inhibited Cdk5 and CD36 Gene Expression Levels in AGE-Exposed U937 Cells

**Figure 3:**
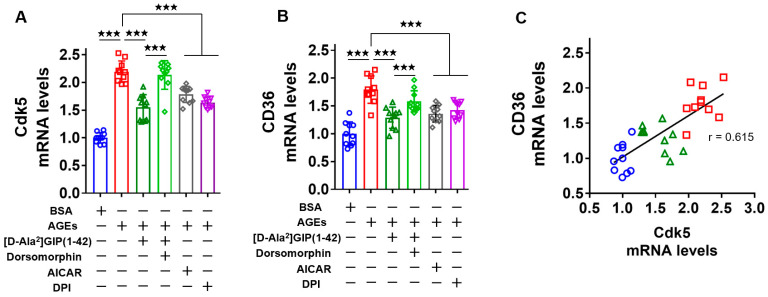
Effects of [D-Ala^2^]GIP(1–42), dorsomorphin, AICAR, and DPI on *Cdk5* and *CD36* gene expression levels in AGE-exposed U937 macrophages. Gene expression levels of *Cdk5* (**A**) or *CD36* (**B**) and their correlation (**C**). The correlation of *Cdk5* with *CD36* gene expression levels was determined by Pearson’s correlation test. Total RNAs were transcribed and amplified by quantitative real-time RT-PCR. The levels of the target gene were initialized by the value of GAPDH mRNA-derived signals and then relatively calculated by the controls with BSA. Number = 10 for each group. Values are presented as mean ± standard deviation. ★★★ *p* < 0.005. (**C**) Blue circles for BSA-treated cells, red squares for AGE-treated cells, and green triangles for AGE-treated cells with [D-Ala^2^]GIP(1–42).

We then elucidated the effects of [D-Ala^2^]GIP(1–42) on *Cdk5* and *CD36* gene expression in AGE-exposed U937 macrophages. As shown in Figure 3A,B, both *Cdk5* and *CD36* gene expression levels were increased in U937 cells by the treatment with AGEs. [D-Ala^2^]GIP(1–42) inhibited the AGE-induced up-regulation of *Cdk5* and *CD36* mRNA levels in U937 cells, which were partly attenuated by dorsomorphin. AICAR mimicked the effects of [D-Ala^2^]GIP(1–42) on *Cdk5* and *CD36* gene expression in AGE-exposed U937 cells. DPI significantly inhibited the AGE-induced up-regulation of *Cdk5* and *CD36* mRNA levels in U937 cells. The levels of *Cdk5* and *CD36* gene expression were highly correlated with each other (Figure 3C).

### 2.4. Correlation of Intracellular ROS Generation with Cdk5 and CD36 Gene Expression, and Association of CD36 mRNA Levels with Dil-ox-LDL Uptake into U937 Cells

**Figure 4:**
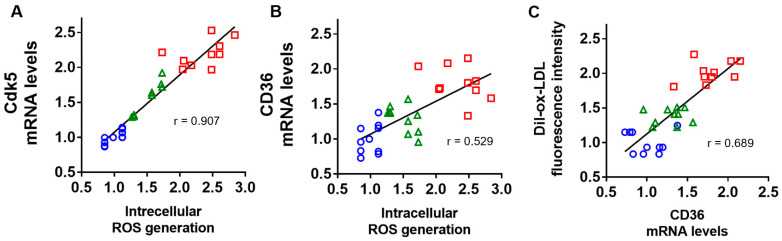
Correlation of intracellular ROS generation with *Cdk5* (**A**) or *CD36* (**B**) mRNA levels and the association of *CD36* gene expression levels with Dil-ox-LDL uptake (**C**) into U937 cells. The correlation was determined by Pearson’s correlation test. Number = 10 for each group. Blue circles for BSA-treated cells, red squares for AGE-treated cells, and green triangles for AGE-treated cells with [D-Ala^2^]GIP(1–42).

We then studied the correlation of intracellular ROS generation with *Cdk5* and *CD36* mRNA levels and the association of *CD36* gene expression levels with ox-LDL uptake. As shown in Figure 4A,B, intracellular ROS generation was correlated with *Cdk5* and *CD36* gene expression levels. CD36 mRNA levels were also positively associated with Dil-ox-LDL uptake into U937 cells (Figure 4C).

## 3. Discussion

A growing body of evidence has shown that the interaction of AGEs, senescent macromolecules formed at an accelerated rate under diabetes, with the cell surface receptor RAGE, evokes oxidative stress generation and inflammatory reactions in numerous kinds of cells, thereby being involved in the development and progression of atherosclerotic cardiovascular disease [23,34,39]. Indeed, AGE accumulation was increased in macrophage foam cells within human atherosclerotic plaque, whose levels were associated with plaque instability [32,33,34]. Foam cell formation and *CD36* gene expression were enhanced in monocyte-driven macrophages extracted from patients with diabetes mellitus in comparison to those from non-diabetic healthy subjects [40]. In addition, we have shown that AGEs significantly enhance macrophage foam cell formation, which was evaluated by ox-LDL uptake into human monocyte-derived macrophages [40,41]. In this previous report, we found that (1) an anti-oxidant, *N*-acetylcysteine, significantly inhibited the AGE-induced foam cell formation and up-regulation of *Cdk5* and *CD36* mRNA levels in U937 cells; (2) a selective inhibitor of Cdk5 suppressed the AGE-induced increase in *CD36* gene expression as well as the foam cell formation of macrophages, (3) the anti-CD36 neutralizing antibody inhibited the foam cell formation of AGE-exposed macrophages, and (4) the blockade of the AGE-RAGE interaction by RAGE-aptamer inhibited the AGE-induced ROS generation, and *Cdk5* and *CD36* gene expression in, and foam cell formation of, U937 macrophages [41]. These observations suggest that the engagement of RAGE with AGEs could contribute to macrophage foam cell formation by stimulating the CD36-dependent ox-LDL uptake into U937 cells via the ROS-Cdk5 signaling pathway.

Tirzepatide, a dual GIP and GLP-1 receptor agonist, has recently been approved for the treatment of patients with diabetes [9,10,11]; it has resulted in superior HbA1c reduction in diabetes patients and exhibited unprecedented weight loss in obese individuals compared with GLP-1 receptor agonists alone. Although several clinical trials have revealed that GLP-1 receptor agonists reduce the risk of cardiovascular disease in patients with diabetes [42,43,44], the effect of the GIP receptor agonist, including tirzepatide, on atherosclerotic cardiovascular disease remains unclear. Therefore, in the present study, we investigated the effect of GIP on AGE-induced macrophage foam cell formation, an initial step of atherosclerosis [21,22,45,46]. The salient findings in the present study were as follows: (1) the *Gipr* and *Rage* gene were expressed in human-cultured U937 macrophages; (2) AGEs increased oxidative stress generation and the foam cell formation of U937 macrophages, both of which were significantly inhibited by their treatment with [D-Ala^2^]GIP(1–42); (3) the anti-atherosclerotic and anti-oxidative effects of [D-Ala^2^]GIP(1–42) on AGE-exposed U937 cells were partly attenuated by an AMPK inhibitor, dorsomorphin; (4) an activator of AMPK, AICAR, mimicked the effects of [D-Ala^2^]GIP(1–42) on foam cell formation and oxidative stress generation in AGE-exposed U937 cells, (5) an inhibitor of NADPH oxidase, DPI, significantly inhibited the AGE-induced increase in foam cell formation and ROS generation in U937 cells; (6) foam cell formation and oxidative stress generation was correlated with each other; (7) AGEs increased *Cdk5* and *CD36* gene expression levels, both of which were significantly blocked by [D-Ala^2^]GIP(1–42); (8) AICAR mimicked the inhibitory effects of [D-Ala^2^]GIP(1–42) on *Cdk5* and *CD36* mRNA levels in AGE-exposed U937 cells, while dorsomorphin partly attenuated the effects of [D-Ala^2^]GIP(1–42) on the AGE-induced up-regulation of these gene levels in U937 cells; (9) DPI significantly inhibited the AGE-induced increase in *Cdk5* and *CD36* gene expression levels in U937 cells; and (10) there was a significant correlation between *Cdk5* and *CD36* gene expression levels in U937 cells. Taken together, the present findings suggest that [D-Ala^2^]GIP(1–42) blocks the AGE-induced foam cell formation of macrophages by inhibiting the Cdk5-CD36 pathway via the suppression of NADPH oxidase-derived ROS generation. 

In this study, we showed, for the first time, that an inhibitor of NADPH oxidase, DPI, inhibited the AGE-induced ROS generation of U937 macrophages, which was associated with the downregulation of *Cdk5* and *CD36* mRNA levels and the subsequent foam cell formation of U937 macrophages. The present observations with our previous findings suggest that NADPH oxidase-derived ROS generation, enhanced by the AGE-RAGE interaction, could stimulate ox-LDL uptake into U937 cells and play a principal role in macrophage foam cell formation through the induction of CD36, one of the major scavenger receptors that can mediate the uptake of ox-LDL, through the activation of Cdk5. We, along with others, have previously reported that NADPH oxidase-derived ROS generation is required for the signaling pathway of the AGE-RAGE axis in various types of cells, including macrophages [47,48], thus suggesting that NADPH oxidase activity and ROS generation may contribute to atherosclerotic foam cell formation in diabetes. 

In the present study, we found that an AMPK inhibitor, dorsomorphin, significantly attenuated the effects of [D-Ala^2^]GIP(1–42) on foam cell formation, ROS generation, and *Cdk5* and *CD36* gene expressions in AGE-exposed U937 cells, whereas an activator of AMPK, AICAR, mimicked the anti-oxidative and anti-atherosclerotic effects of [D-Ala^2^]GIP(1–42). We have previously shown that metformin inhibited AGE-RAGE-induced proliferation and *vascular endothelial growth factor* gene expression in breast cancer cells, both of which were blocked by compound C, an inhibitor of AMPK [49]. Moreover, metformin has been shown to exert anti-oxidative effects on lipopolysaccharide-exposed macrophages via the activation of AMPK [50]. In addition, globular adiponectin has been reported to inhibit ethanol-induced ROS generation in macrophages by suppressing NADPH oxidase activity via the AMPK pathway [51]. These findings suggest that NADPH oxidase-derived ROS generation may be a molecular target of the anti-atherosclerotic effects of [D-Ala^2^]GIP(1–42) on U937 macrophages via AMPK activation. In other words, [D-Ala^2^]GIP(1–42) may inhibit the AGE-RAGE-induced oxidative stress-mediated macrophage foam cell formation by suppressing NADPH oxidase activity through its interaction with the GIP receptor via the activation of the AMPK pathway. Several studies have shown that the binding of GIP with the GIP receptor activates AMPK through phospholipase C and calcium/calmodulin-dependent protein kinase signaling pathways [15,52,53,54], thus supporting the concept that AMPK could play a role in the anti-atherosclerotic effects of [D-Ala^2^]GIP(1–42) observed here.

NADPH oxidase activity in macrophages was inhibited by the cyclic AMP–protein kinase A pathway [55]. We previously showed that GIP, a stimulator of cyclic AMP or an inhibitor of NADPH oxidase, inhibits AGE-RAGE-induced oxidative stress generation and inflammatory and thrombotic reactions in endothelial cells [16]. Given that GIP stimulates cyclic AMP production in a variety of cells [16,56,57], [D-Ala^2^]GIP(1–42) may inhibit AGE-induced macrophage foam cell formation by stimulating the cyclic AMP-protein kinase A pathway via the suppression of NADPH oxidase activity. The effects of [D-Ala^2^]GIP(1–42) on foam cell formation, ROS generation, and *Cdk5* and *CD36* gene expressions in U937 cells exposed to AGEs were not completely inhibited by the co-treatment of dorsomorphin, thus suggesting that components other than the AMPK pathway may also be involved in the anti-atherosclerotic action of [D-Ala^2^]GIP(1–42). 

We have recently found that *Stachybotrys microspore* triprenyl phenol-44D inhibits atherosclerotic plaque lesions in *Apoe*^−/−^ mice by attenuating AGE-RAGE-induced macrophage foam cell formation through the Cdk5-CD36 pathway suppression [58]. The observations suggest that accelerated macrophage foam cell formation within atherosclerotic plaques may be a therapeutic strategy for preventing atherosclerotic cardiovascular disease. Therefore, our present study suggests that the GIP-GIP receptor axis may exert anti-atherosclerotic actions by suppressing AGE-RAGE-induced foam cell formation in diabetes. Further clinical studies are needed to clarify whether GIP-based therapy could actually reduce the risk of cardiovascular events in diabetes patients.

**Figure 5:**
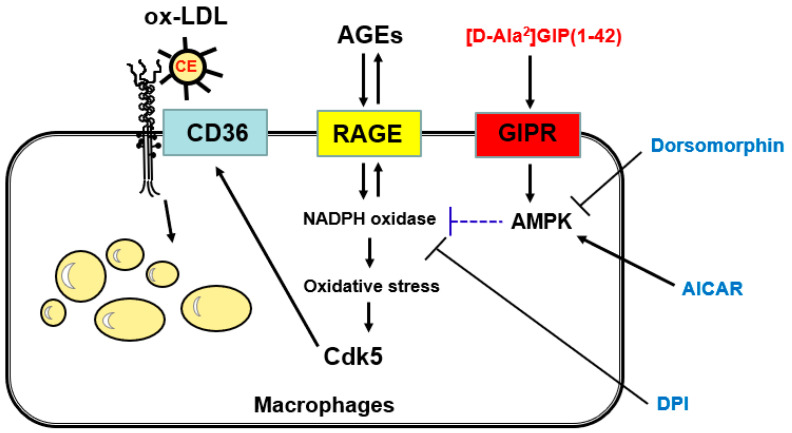
Possible mechanisms of the inhibitory effect of [D-Ala^2^]GIP(1–42) on foam cell formation of macrophages. AGEs, advanced glycation end products; RAGE, receptor for AGE; GIPR, receptor of glucose-dependent insulinotropic polypeptide; AMPK, AMP-activated protein kinase; Cdk5, cyclin-dependent kinase 5; CE, cholesterol ester; DPI, diphenylene iodonium; AICAR, 5-aminoimidazole-4-carboxamide1-B-D-ribofuranoside; and ox-LDL, oxidized low-density lipoprotein.

There were several limitations in the present experiments. First, we did not evaluate the protein expression levels of RAGE, Cdk5, and CD36 in this study. To examine these protein expression levels would be helpful to support the findings of this study. However, CD36 protein expression levels are reported to be functionally correlated with ox-LDL uptake and the foam cell formation of macrophages [59]. We have previously found that the gene expression levels of *Cdk5* and *CD36* are correlated with each other in AGE-exposed U937 macrophages [41]. Moreover, we have shown that the blockade of the AGE-RAGE signaling pathway by *N*-acetyl-l-cysteine or RAGE-aptamer reduces *Cdk5* mRNA levels and suppresses *CD36* gene expression and the foam cell formation of macrophages, the latter of which is also suppressed by an inhibitor of Cdk5 [41]. Therefore, the present and previous observations suggest that gene expressions of *RAGE*, *Cdk5*, *CD36* can be correlated with each protein expression, respectively. Second, we did not exactly know how Cdk5 regulated CD36 expression in U937 macrophage cells. However, Cdk5 might increase the phosphorylation of peroxisome proliferator-activated receptor γ and subsequently enhance its transcriptional activity, which could cause CD36 overexpression and macrophage foam cell formation [41,60,61]. Third, we chose the concentration of AGE-BSA at 100 μg/mL in this study because its levels were comparable with those of diabetic patients [62]. Fourth, in this study, we evaluated macrophage foam cell formation by Dil-ox-LDL uptake. We examined the involvement of NADPH oxidase in the macrophage foam cell formation exposed by AGEs because we previously showed that the AGE-RAGE interaction stimulates NADPH oxidase activity in endothelial cells, the pathological effect of which was blocked by an inhibitor of NADPH oxidase activity, DPI [47]. Therefore, we did not examine intracellular levels of NADPH, endothelial nitric oxide synthase, and other anti-oxidant systems in the present study. Fifth, although NADPH oxidase activation may have dual effects in diabetes, there is a growing body of evidence that the inhibition of NADPH oxidase activity ameliorates vascular injury and metabolic derangement in diabetes [63,64]. Sixth, we did not investigate the tissue-specific distribution of various NAD(P)H oxidase subunits and distinctive functions of Nox isoforms. However, we have previously shown that the AGE-RAGE axis up-regulates the gene expression of all the components of NADPH oxidase, including *gp91^phox^*, *Nox1*, and *Nox4*, in diabetic kidneys [39]. It would be interesting to examine the effects of [D-Ala^2^]GIP(1–42) on the expression of these NADPH oxidase components in macrophages exposed to AGEs (Figure 5).

## 4. Materials and Methods

### 4.1. Chemical Regents and Materials

[D-Ala2]GIP(1–42) was purchased from Phoenix Pharmaceuticals. Inc. (Burlingame, CA, USA). Dil-ox-LDL was acquired from Highland Technology Center (Frederick, MD, USA), and an OxiSelect intracellular ROS assay kit and DCFH-DA probe were acquired from Cell Biolabs. Inc. (San Diego, CA, USA). A human cell line of monocytes, U937 cells, were obtained from the Japanese Collection of Research Bioresources Cell Bank (JCRB9021; Osaka, Japan); the AMPK inhibitor, dorsomorphin, was obtained from FUJIFILM Wako Pure Chemical Corporation (Osaka, Japan); and an inhibitor of NADPH-oxidase, DPI, an AMPK stimulator AICAR, phorbol 12-myristate 13-acetate (PMA), D-glyceraldehyde, Roswell Park Memorial Institute (RPMI) 1640 medium, BSA, and fetal bovine serum (FBS) were obtained from Sigma Aldrich (St. Louis, MO, USA). A Vibrance Antifade Mounting Medium with DAPI was acquired from Vector Laboratories (Burlingame, CA, USA).

### 4.2. Preparation of AGEs of Bovine Serum Albumin (BSA) 

BSA (25 mg/mL) was incubated with 0.1 mol/L D-glyceraldehyde in a 0.2 M NaPO_4_ buffer at 37 °C under sterile conditions for 7 days, as described previously [40,41,47,49,58]. Then, unincorporated sugars were removed using dialysis against phosphate-buffered saline (PBS) to prepare AGEs [40,41,47,49,58]. In the same conditions, except for the absence of D-glyceraldehyde, non-glycated BSA was prepared and used as a control.

### 4.3. Cell Culture of U937 Monocyte-Derived Macrophages

U937 cells were maintained in the RPMI 1640 medium with 10% FBS, as described previously [18,40,41]. The cells were seeded onto 12 well plates at a concentration of 1.0 × 10^6^ cells/dish and cultured with PMA (40 ng/mL) in the same medium. After washing with PBS, adherent cells were used as differentiated U937 macrophages [18,41]. 

### 4.4. Measurement of Fluorescence Intensity of Dil-ox-LDL and ROS Generation in U937 Cells

U937 macrophage cells were incubated with 100 μg/mL AGE-BSA or 100 μg/mL non-glycated BSA in the presence or absence of 1 nmol/L [D-Ala^2^]GIP(1–42) [18], 50 nmol/L DPI, an inhibitor of NADPH oxidase [47], 10 μmol/L dorsomorphin, an inhibitor of AMPK [15], and 500 μmol/L AICAR, an activator of AMPK [15] in RPMI 1640 medium supplemented with 10% FBS for 24 h [18,40,41]. After removing the cell medium, cells were treated with 10 μg/mL Dil-ox-LDL for 18 h and with a 10 μmol/L DCFH-DA probe [65,66] for another 15 min. Then, adherent U937 macrophages were mounted in a Vibrance Antifade Mounting Medium with DAPI, and immunofluorescence images were obtained using the All-in-One Fluorescence Microscope BZ-X710 and Analysis Software BZ-X800 (Keyence; Osaka, Japan). The fluorescence intensity area, of red or green color, per the cells was calculated as described previously [18,40,41,58]. 

### 4.5. Quantitative Real-Time Reverse Transcription–Polymerase Chain Reaction (RT-PCR)

Total RNA was extracted from the adherent human U937 cells to synthesize cDNA. The SYBR Green or TaqMan-based gene expression assay and StepOnePlus^TM^ sequence detection system (Life Technologies Japan, Tokyo, Japan) were used for quantitative real-time RT-PCR. The expression levels of target genes were initialized by the values of glyceraldehyde 3-phosphate dehydrogenase (GAPDH) and then related to the controls with BSA, as described previously [18,40,41,58]. The primers and probes used for the analysis of quantitative RT-PCR were as follows: Gipr, Hs00609201_g1; Rage, Hs00542584_g1; Cdk5, NM_001164410.3, NM_004935.4; CD36, Hs00169627_ml; and Gapdh, Hs99999905_ml.

### 4.6. Statistical Analysis

All data are expressed as the mean ± standard deviation. The parametric coefficient of Pearson’s correlation was used to analyze the correlation between the two groups. GraphPad PRISM ver. 7.05 software (GraphPad Inc., San Diego, CA, USA) was used for statistical analysis. The significance level was defined as a *p*-value < 0.05.

## 5. Conclusions

The present study suggests that [D-Ala^2^]GIP(1–42) could inhibit AGE-RAGE-induced, NADPH oxidase-derived oxidative stress generation in U937 macrophages via AMPK activation and subsequently suppress macrophage foam cell formation by reducing the Cdk5-CD36 pathway.

## Data Availability

Data are contained within the article.
